# Nonsteroidal Anti-Inflammatory Drugs Reduce Second Cancer Risk in Patients With Breast Cancer: A Nationwide Population-Based Propensity Score-Matched Cohort Study in Taiwan

**DOI:** 10.3389/fonc.2021.756143

**Published:** 2021-11-24

**Authors:** Yin-Che Lu, Pin-Tzu Chen, Mei-Chen Lin, Che-Chen Lin, Shi-Heng Wang, Yi-Jiun Pan

**Affiliations:** ^1^ Division of Hematology–Oncology, Ditmanson Medical Foundation Chia-Yi Christian Hospital, Chia-Yi, Taiwan; ^2^ Department of Nursing, Min-Hwei Junior College of Health Care Management, Tainan, Taiwan; ^3^ Management Office for Health Data, China Medical University Hospital, Taichung, Taiwan; ^4^ School of Medicine, China Medical University, Taichung, Taiwan; ^5^ Department of Occupational Safety and Health, China Medical University, Taichung, Taiwan; ^6^ Department of Public Health, China Medical University, Taichung, Taiwan

**Keywords:** NSAID, breast cancer, second cancer, cohort study, risk reduction

## Abstract

Nonsteroidal anti-inflammatory drugs (NSAIDs) reduce mortality in patients with cancer, especially breast cancer, but their influence on second cancer risk is uncertain. This study aimed to examine whether NSAID use is associated with second cancer risk in patients with breast cancer. This population-based propensity score-matched cohort study using Taiwan’s National Health Insurance Research Database enrolled patients with newly diagnosed breast cancer (n = 7356) with and without (n = 1839) NSAID therapy from 2000 to 2009. They were followed up until the diagnosis of second cancer, death, or end of 2011. Cox proportional hazard models were used to estimate adjusted hazard ratios (aHR). The NSAID cohort had a lower incidence rate of second cancer than the non-NSAID cohort (5.57 *vs*. 9.19 per 1,000 person-years), with an aHR of 0.63 (95% confidence interval (CI) 0.46–0.87). When compared with the non-NSAID cohort, the second cancer incidence was lower in patients taking non-cyclooxygenase 2 inhibitors (aHR 0.67, 95% CI 0.47–0.94) and in those receiving multiple NSAIDs during follow-up (aHR 0.55, 95% CI 0.37–0.84). A dose–response relationship existed in NSAID cumulative days. The findings demonstrate that NSAID use reduces second cancer risk in a dose-dependent manner in patients with primary breast cancer.

## Introduction

Breast cancer is one of the rapidly growing cancers in developed countries. In Taiwan, with the increase in breast cancer incidence and its associated medical-resource utilization (1), the chance of early breast cancer detection has increased significantly owing to the implementation of the national policy on mammography screening (2). In addition to resection surgery, patients with breast cancer usually receive adjuvant chemotherapy, radiotherapy, and hormone therapy after surgery. Chemotherapeutic drugs, hormone-based drugs, and radiation contribute to the long-term survival benefit of patients with breast cancer. However, these treatments have some inevitable long-term side effects, especially the increased risk of second cancer development (3). Therefore, with the increase in the long-term survival of patients with breast cancer, the problem of second cancer should be focused on during the follow-up of long-term survivors.

Nonsteroidal anti-inflammatory drugs (NSAIDs) include aspirin and cyclooxygenase (COX) inhibitors. COX inhibitors are further divided into COX-2 selective and non-selective (non-COX-2) classes. In recent years, aspirin and NSAIDs have been suggested to have inhibitory effects on the pathogenesis of cancerization (4, 5), and evidence in this regard has been accumulating from basic and clinical research (6–9). Some studies have revealed that the use of aspirin or NSAIDs reduce the risk of breast cancer in the general population (10, 11) and in individuals with a family history of breast cancer (12). This cancer-preventive effect has also been observed in certain populations such as patients with chronic dialysis (13), women with obesity (14), and persons without smoking habits (15). Furthermore, reduced cancer-related mortality has been shown in patients with cancer using NSAIDs (16–18). Especially, the use of NSAIDs after breast cancer diagnosis could reduce mortality in patients with breast cancer (19–21). However, to the best of our knowledge, no study has so far examined whether NSAID use has any effect on second cancer risk in patients with breast cancer.

Performing randomized clinical trials with NSAIDs is challenging because these are old patent-off drugs. Conducting clinical trials is a time-consuming and expensive affair. Unlike new anti-cancer drugs, recruiting a sufficient number of patients to participate in NSAID-related clinical trials is difficult. The Taiwan National Health Insurance Research Database (NHIRD) has collected rich information on drug use history, hospitalization history, and disease event in Taiwanese patients over the past 20 years (22), which serves as a data resource for biomedical research (23). This study aimed to investigate the association of NSAID use with the incidence of second cancer in patients with primary breast cancer by using the NHIRD data.

## Materials and Methods

### Data Sources

We collected data from the NHIRD, derived from Taiwan’s single-payer compulsory National Health Insurance Program, which covers up to 99% of the 23 million Taiwanese citizens. The NHIRD contains inpatient, and outpatient visit dates, medical diagnosis, expenditure, and prescription details. This retrospective cohort study used the Registry of Catastrophic Illness Patient Database (RCIPD), a part of the NHIRD. The insurants in the RCIPD were individuals with fatal illnesses, including 30 categories of diseases requiring long-term extensive care, such as cancer. The disease record system in the NHIRD was built based on the International Classification of Disease, Ninth Revision, Clinical Modification (ICD-9-CM). This study was approved by the Research Ethics Committee of China Medical University (CMUH-104-REC2-115-R4), and the need for informed consent was waived. This study adheres to the Strengthening the Reporting of Observational Studies in Epidemiology (STROBE) Statement: guidelines for reporting observational studies.

### Study Subjects

A population-based cohort study was conducted to explore the association of NSAID use with second cancer risk in patients with primary breast cancer. We established a breast cancer cohort of 64,263 women (aged ≥20 years) with newly diagnosed breast cancer (ICD-9-CM 174) from 2000 to 2009.

**Figure 1:**
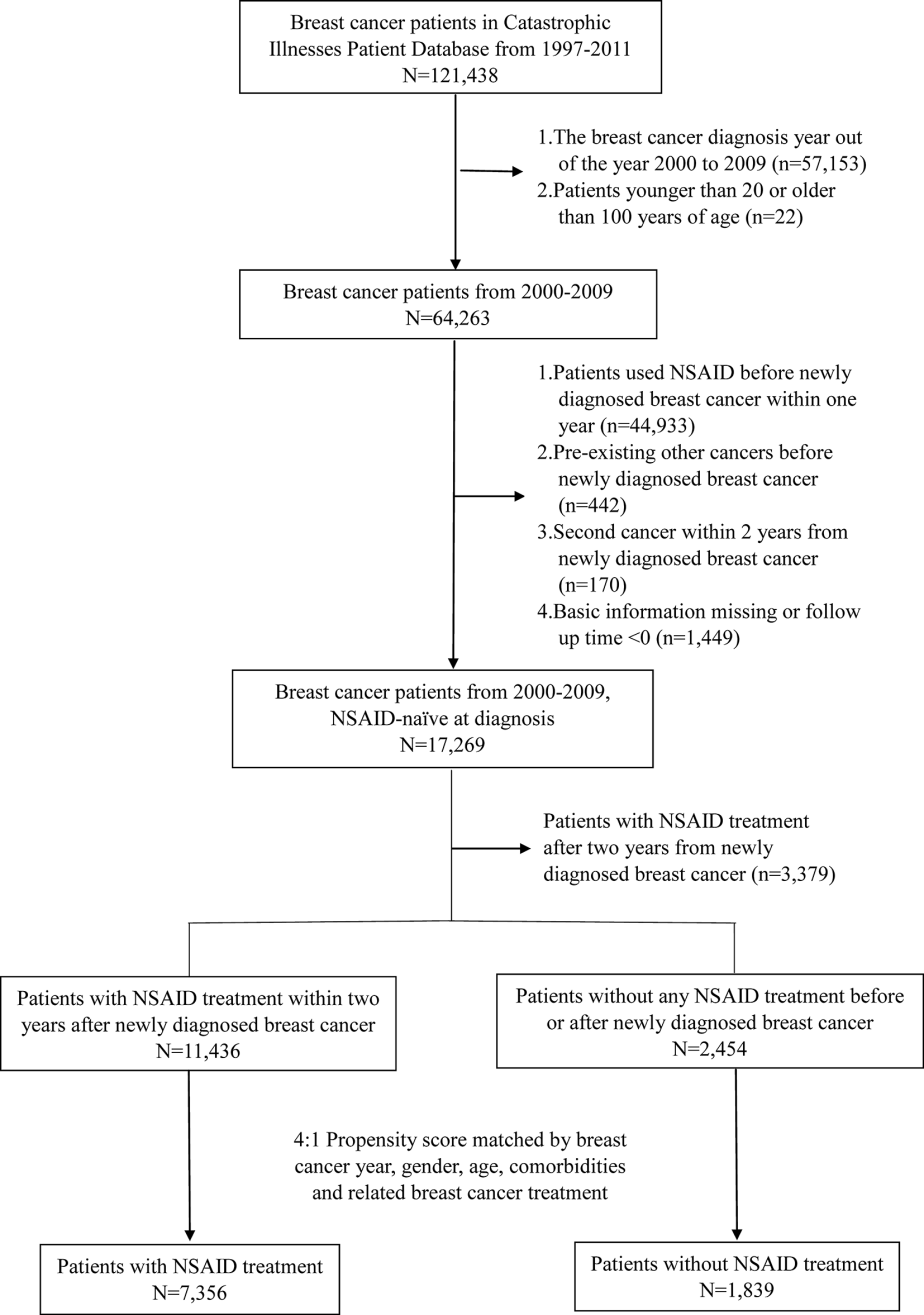
Flowchart of the propensity score-matched cohort study.

The exclusion criteria of the study were as follows: preexisting cancers other than breast cancer, history of using NSAIDs before the diagnosis of breast cancer, and second cancer within 2 years of newly diagnosed breast cancer. Cancer takes time to develop; hence, we determined that the second cancer must occur at least two years after the primary breast cancer. This definition was intended to clearly define that these two events did not occur simultaneously. A total of 17,269 patients with newly diagnosed breast cancer who were NSAID-naïve at diagnosis and did not have other preexisting cancers were selected. Patients with NSAID treatment 2 years after the newly diagnosed breast cancer were further excluded (n = 3,379). In this study, the NSAID cohort was defined as patients with NSAID treatment within 730 days (2 years) of the initial date of breast cancer diagnosis to avoid immortal time. Day 731 after the diagnosis date of breast cancer was defined as the index date. The flowchart is shown in **Figure 1**.

### Covariates

The distribution of age and baseline comorbidities was compared between patients with and without NSAID treatment (*n* = 11,436 and 2,454, respectively) (**Table 1**). Sixteen baseline comorbidities were identified in our study, including congestive heart failure (ICD-9-CM codes 398.91, 425, and 428), dementia (ICD-9-CM codes 290.0-290.4, 294.1, and 331.0-331.2), chronic obstructive pulmonary disease (ICD-9-CM codes 491, 492, and 496), rheumatic disease (ICD-9-CM odes 710, 714, 725, and A430), peptic ulcer disease (ICD-9-CM codes 531-534), cirrhosis (ICD-9-CM codes 571, A347), hypertension (ICD-9-CM codes 401-405, A260, and A269), diabetes (ICD-9-CM code 250), hypercholesterolemia (ICD-9-CM code 272.0), hyperlipidemia (ICD-9-CM codes 272.2 and 272.4), ischemic heart disease (ICD-9-CM codes 410-414 and 429), atrial fibrillation and flutter (ICD-9-CM code 427.3), cardiovascular disease (ICD-9-CM codes 410-414, 428, 430-438, and 440-448), peripheral vascular disease (ICD-9-CM code 440-444), chronic kidney disease (ICD-9-CM codes 580-589 and A350), and depression (ICD-9-CM codes 296.2, 296.3, 296.82, 300.4, 309.0, 309.1, 309.28, and 311). The criterion of at least one inpatient or two outpatient admissions for such diseases before the initial date of breast cancer diagnosis was used to enhance diagnostic precision.

**Table 1:** Distribution of age, comorbidity, breast cancer-related treatment, and second cancer between the non-NSAID and NSAID cohorts.

	Before PSM			After 1:4 PSM		
Variable	NSAID non-users	NSAID users	p-value	NSAID non-users	NSAID users	p-value
	N = 2454 (17.7%)	N = 11436 (82.3%)		N = 1839 (20.0%)	N = 7356 (80.0%)	
**Age, years**			0.014			0.430
<40	761 (31)	3641 (31.8)		592 (32.2)	2485 (33.8)	
40–64	1457 (59.4)	6492 (56.8)		1062 (57.7)	4155 (56.5)	
≥65	236 (9.6)	1303 (11.4)		185 (10.1)	716 (9.7)	
Mean (SD)	50.6 (10.8)	50.7 (11.1)	0.753	50.4 (11.0)	49.9 (10.7)	0.061
**Comorbidity**						
Congestive heart failure	31 (1.3)	201 (1.8)	0.083	29 (1.6)	106 (1.4)	0.665
Dementia	21 (0.9)	66 (0.6)	0.112	11 (0.6)	40 (0.5)	0.779
Chronic obstructive pulmonary disease	124 (5.1)	927 (8.1)	<0.001	119 (6.5)	440 (6.0)	0.432
Rheumatic disease	103 (4.2)	675 (5.9)	0.001	92 (5.0)	345 (4.7)	0.573
Peptic ulcer	368 (15)	2446 (21.4)	<0.001	348 (18.9)	1304 (17.7)	0.232
Liver Cirrhosis	380 (15.5)	2266 (19.8)	<0.001	325 (17.7)	1240 (16.9)	0.405
Hypertension	435 (17.7)	2662 (23.3)	<0.001	383 (20.8)	1421 (19.3)	0.145
Diabetes mellitus	228 (9.3)	1381 (12.1)	<0.001	190 (10.3)	744 (10.1)	0.782
Hyperlipidemia	71 (2.9)	363 (3.2)	0.468	56 (3.0)	197 (2.7)	0.389
Hypercholesterolemia	240 (9.8)	1499 (13.1)	<0.001	216 (11.7)	807 (11.0)	0.345
Ischemic heart disease	147 (6)	1074 (9.4)	<0.001	132 (7.2)	488 (6.6)	0.406
Atrial fibrillation	57 (2.3)	287 (2.5)	0.589	42 (2.3)	171 (2.3)	0.917
Cerebrovascular disease	225 (9.2)	1600 (14)	<0.001	210 (11.4)	764 (10.4)	0.198
Peripheral vascular disease	29 (1.2)	194 (1.7)	0.066	26 (1.4)	95 (1.3)	0.681
Chronic kidney disease	123 (5)	717 (6.3)	0.018	96 (5.2)	389 (5.3)	0.907
Depression	103 (4.2)	693 (6.1)	<0.001	98 (5.3)	338 (4.6)	0.185
**Breast cancer-related treatment**						
Chemotherapy	833 (33.9)	4364 (38.2)	<0.001	728 (39.6)	2941 (40.0)	0.758
Hormone therapy	1694 (69)	8298 (72.6)	<0.001	1353 (73.6)	5336 (72.5)	0.374
Target therapy	146 (5.9)	856 (7.5)	0.008	135 (7.3)	535 (7.3)	0.920
Surgery	2160 (88)	10468 (91.5)	<0.001	1697 (92.3)	6803 (92.5)	0.767

PSM, propensity score matching.

Breast cancer-related treatments, including chemotherapy, hormone therapy, target therapy, and surgery, were also identified. Chemotherapy was defined as the administration of one of the following drugs: docetaxel, paclitaxel, doxorubicin, and epirubicin, which are essential components in various chemotherapeutic regimens. Hormone therapy was defined as the administration of one of the following hormone-based drugs: tamoxifen, anastrozole, letrozole, and exemestane. Target therapy was defined as treatment with trastuzumab. Surgical treatment was defined as having undergone any of the following procedures: partial mastectomy (ICD-9-CM code 85.2-85.25), total mastectomy, and modified radical mastectomy (ICD-9-CM code 85.4-85.48).

### Study Cohorts With Propensity Score Matching (PSM)

Patients with NSAID therapy within 2 years were older and had a higher proportion of baseline comorbidity, chemotherapy, hormone therapy, target therapy, and surgery than those without NSAID therapy (**Table 1**). In this study, 1:4 PSM was performed to identify the non-NSAID cohort. The propensity score was estimated by logistic regression for the controlling of breast cancer year, age, and baseline comorbidities. Following PSM, 7,356 patients with and 1,839 patients without NSAID treatment were included in this study. Both cohorts were followed up until the occurrence of a secondary cancer (ICD-9-CM 140–208, except for breast cancer), death, or end of 2011.

In the NSAID cohort, NSAID products and their dosage were retrieved based on the treatment history during the follow-up. Three different NSAIDs were specified in the study cohort, namely, aspirin, COX-2 inhibitors, and non-COX-2 inhibitors. The NSAID dosage was grouped based on cumulative days: <7, 8–28, 29–90, and >90 days.

### Statistical Analysis

Categorical variables were presented as numbers (percentage), and continuous variables were expressed as means (standard deviation). Cox proportional hazards regression model analyses were performed to assess the hazard ratio (HR) of second cancer for the NSAID cohort in comparison with the non-NSAID cohort. Death could be a competing risk for second cancer; hence, competing risk analysis was conducted by using Fine and Gray’s proportional hazards regression analysis (24) to estimate the subdistribution HR (SHR) and 95% confidence interval (CI). Age- and comorbidity-stratified SHR of second cancer were also estimated for the NSAID cohort. Data management and statistical analyses were performed using SAS software 9.4 (SAS Institute, Cary, NC, USA). The significance level was set at *P* < 0.05 using two-tailed tests.

## Results

**Figure 2:**
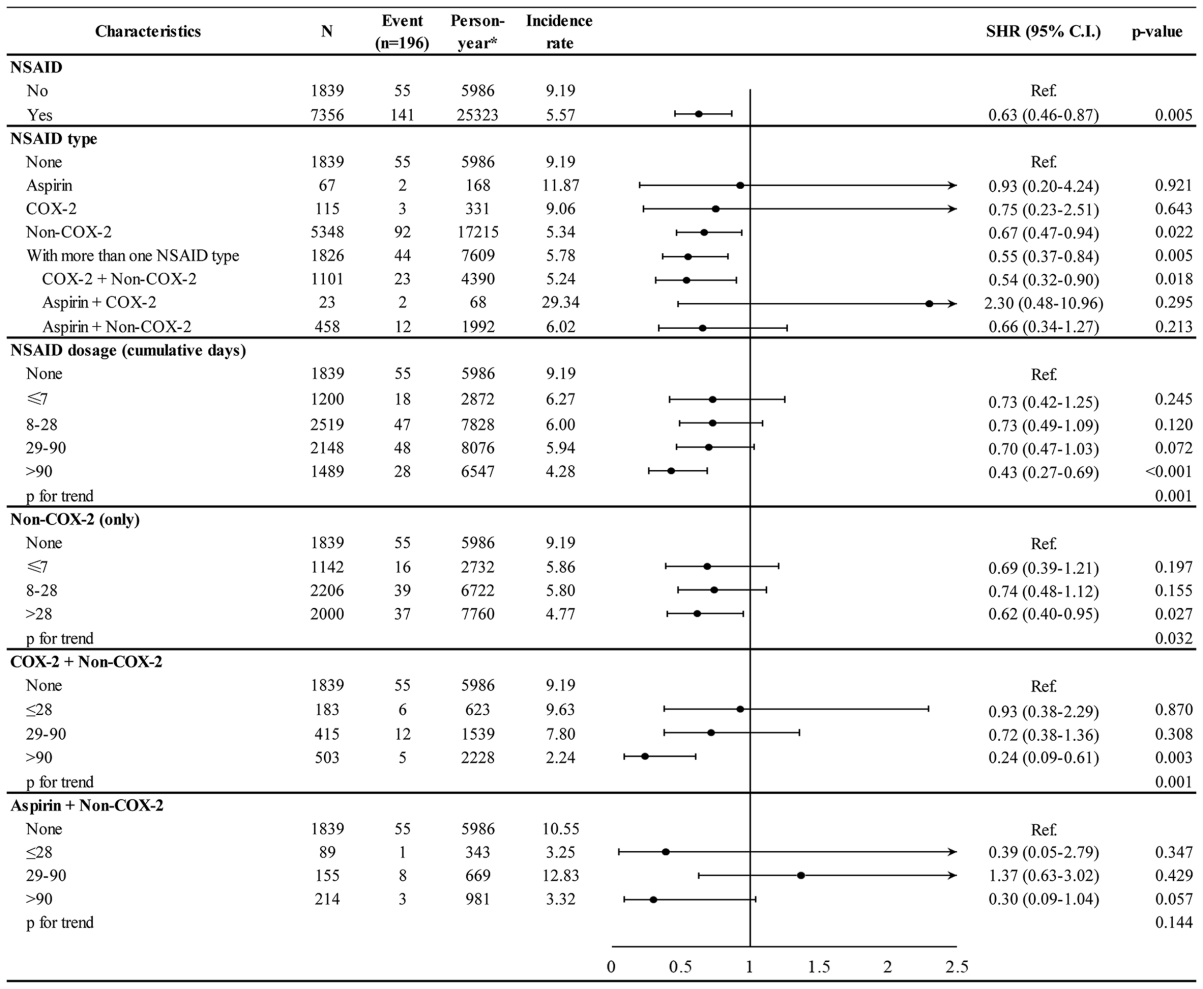
Incidence and hazard ratio of second cancer for NSAID treatment, NSAID type, and dosage.

After the PSM, the baseline characteristics were well balanced between the NSAID and non-NSAID cohorts (**Table 1**). The incidence rate per 1,000 person-years for second cancer was 5.57 in the NSAID cohort and 9.19 in the non-NSAID cohort (**Figure 2**). After adjusting for age, comorbidities, and breast cancer-related treatment, the adjusted SHR of NSAID use was 0.63 (95% CI 0.46–0.87) for second cancer incidence.

The association of second cancer risk with NSAID type and dosage is shown in **Figure 2**. When compared with the non-NSAID cohort, the second cancer incidence was significantly lower in patients taking non-COX-2 inhibitors (adjusted SHR 0.67, 95% CI 0.47–0.94) and in those receiving multiple NSAIDs during follow-up (adjusted SHR 0.55, 95% CI 0.37–0.84). Higher cumulative days of NSAID use was associated with a lower incidence of second cancer in a dose–response manner (p for trend 0.001), and the adjusted SHR was 0.43 (95% CI 0.27–0.69) in patients using NSAID for >90 days. The dose–response relationship was also observed for a specific NSAID type. Patients using non-COX-2 inhibitors for >28 days and COX-2 along with non-COX-2 inhibitors for >90 days had a lower incidence of second cancer, with adjusted SHR of 0.62 (95% CI 0.40–0.95) and 0.24 (95% CI 0.09–0.61), respectively.

**Figure 3:**
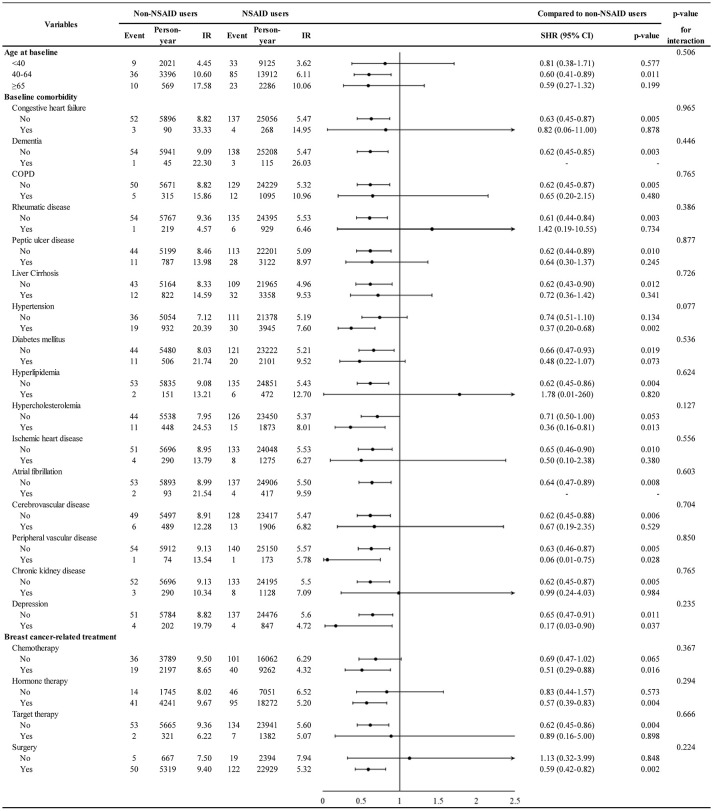
Incidence and hazard ratio of second cancer for NSAID stratified by age, comorbidity, and breast cancer-related treatment.

The association of comorbidity with second cancer is shown in **Supplementary Figure 1**. Patients with liver cirrhosis (adjusted SHR 1.52, 95% CI 1.06–2.18) had a higher HR for second cancer. Furthermore, the association of NSAID treatment with second cancer stratified by age and comorbidities is depicted in **Figure 3**. NSAID treatment was associated with a lower second cancer incidence in patients with hypertension (adjusted SHR 0.37, 95% CI 0.20–0.68) but not in those without hypertension (adjusted SHR 0.74, 95% CI 0.51–1.10), and the hypertension–NSAID interaction reached a borderline significance (p = 0.077).

**Figure 4:**
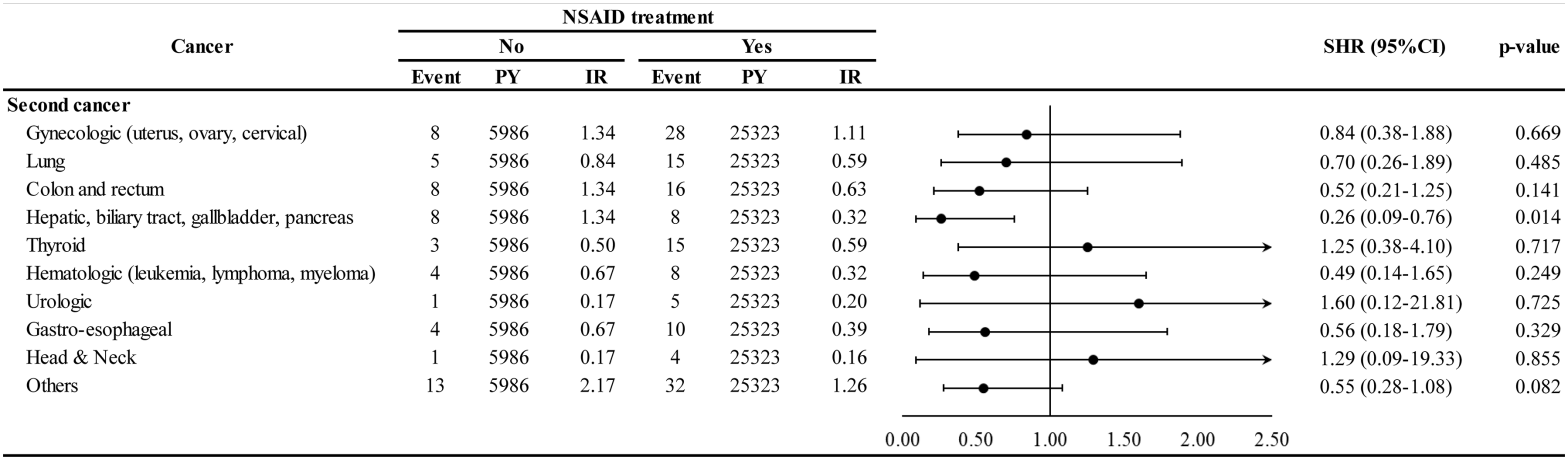
Incidence and hazard ratio of specific second cancer for NSAID. PY, person-years; IR, incidence rate, per 1000-per years; SHR, subdistribution hazard ratio; CI, confidence interval.

The association of NSAID treatment with different types of second cancer is shown in **Figure 4**. The NSAID cohort demonstrated lower HR for hepatic, biliary tract, gallbladder, and pancreatic cancers (adjusted SHR 0.26, 95% CI 0.09–0.76) than the non-NSAID cohort. However, NSAID use did not have a protective effect in case of a subsequent gynecologic cancer (adjusted SHR 0.84, 95% CI 0.38–1.88). The statistical power of such second cancer stratified analyses could be limited because of the small sample size for each specific second cancer.

## Discussion

In this nationwide population-based cohort study of patients with primary breast cancer, PSM was used to establish the NSAID and non-NSAID cohorts that were similar in terms of baseline characteristics, and their subsequent second cancer risk was investigated. This study also evaluated the sub-classifications of NSAIDs, their combined use, and NSAID dosage in cumulative days with respect to the patients’ carcinogenicity. This study showed that the NSAID cohort had a lower incidence of second cancer than the non-NSAID cohort. The risk was particularly lower for patients undergoing non-COX-2 treatments, those receiving non-COX-2 along with COX-2 during the follow-up, and those receiving NSAID dosage for a cumulative period of >90 days.

In our study, drug subgroup analysis unexpectedly revealed that the protective effect of NSAID is often derived from non-COX-2 drugs. The use of aspirin and COX-2 drugs had no lowering effect on the incidence of second cancer in patients with primary breast cancer. Aspirin was the most disputed drug among all NSAIDs. The reason for the increased attention was that aspirin exerted a protective effect in the normal population based on the findings of clinical studies (25–27). However, this drug could not reduce breast cancer-specific mortality in patients with primary breast cancer, as reported by many clinical studies (28–30). Strasser-Weippl et al. also showed that COX-2 combined with aspirin has no effect on the disease-free survival of patients with breast cancer (31). Our study suggested that non-COX-2 and COX-2 drugs have different effects in reducing the incidence of second cancer in patients with breast cancer. Such inconsistent findings might reflect the actual drug consumption frequency among the people in Taiwan (aspirin N = 67, COX-2 N = 115, and non-COX-2 N = 5348). The fact that >90% of the patients were in the non-COX2 cohort made the number of patients in the other two cohorts (aspirin and COX-2) too small to reveal their statistical effect. Furthermore, many studies have shown that non-selective COX-2 drugs have better anti-cancer activity than selective COX-2 agents (12, 32, 33).

Another important study finding was that using more than one NSAID during the follow-up had variable effects in reducing the incidence of second cancer as the use of COX-2 along with non-COX-2 drugs during the follow-up could reduce second cancer risk. However, aspirin combined with either COX-2 or non-COX-2 drug did not reduce second cancer risk. He et al. also found that the use of aspirin combined with COX-2 reduces COX-2 chemopreventive effects in colorectal cancer (34). Additionally, gastrointestinal studies have demonstrated that the concomitant use of aspirin and COX-2 reduces the gastrointestinal benefit of COX-2 (35, 36). Our study reveals that the NSAID dosage is proportional to the reduction in the incidence of second cancer risk. Other studies have also demonstrated that the cancer-preventive effect of NSAID is dose-dependent (7, 13, 16), including an intake duration of at least 90 days (6, 37–39), 180 days (15, 39), or longer (years) (14, 33).

Our study showed that NSAID treatment is associated with a lower second cancer incidence in patients aged 40–64 years (SHR = 0.60, p-value = 0.011) as well as in patients aged >65 years (SHR = 0.59, though not reached statistical significance) (**Figure 3**). This result implies that both pre-menopausal and post-menopausal women with breast cancer can derive chemopreventive benefit by using NSAID. Previous studies have also shown that NSAID exhibits its chemopreventive effect in pre- and post-menopausal women (12, 25, 32); nonetheless, some studies have shown that post-menopausal women could particularly benefit from the NSAID anti-cancer effect (26, 27, 33).

This study observed that liver cirrhosis is associated with increased second cancer incidence (see **Supplementary Figure 1**). Globally, Taiwan was a country with a high prevalence of hepatitis B and hepatitis C (40), which caused a higher incidence of liver cirrhosis and lead to an increased cancer incidence (41, 42). In our study, the protective influence of NSAID on second cancers was more predominant in patients with hypertension. Such patients have been well-educated to take antihypertensive drugs periodically, and previous studies have proposed that antihypertensive drugs might increase cancer risk (43–45). The interactive effect between NSAIDs and antihypertensive drugs needs to be explored further.

Although NSAID use decreased second cancer in many organ systems, only hepatic, biliary tract, and pancreatic cancers showed significance (**Figure 4**). Previous studies in Taiwan have also found that NSAID use decreased hepatocellular carcinoma risk in patients with chronic hepatitis B and infection (6, 16, 37).

A key strength of this study is the novelty of examining the association between NSAID use and second cancer incidence in patients with primary breast cancer. The use of a nationwide representative cohort design with large sample sizes and the application of PSM to consider a range of comorbidities and breast cancer-related treatments and to balance their distributions in the two cohorts with and without NSAID therapy are other important advantages. Furthermore, this study has provided empirical evidence for the association of NSAID use with second cancer risk reduction in a dose–response manner.

However, this study also has several limitations. First, the study is relatively old and has a relatively short study period, i.e., from 1997 to 2011. It takes at least 4–5 years for second cancer to develop in primary breast cancer survivors. We selected women with newly diagnosed breast cancer from 2000 to 2009 and then followed them up in 2011. A longer follow-up period might allow more cases of primary breast cancer to fit into our study and allow more second cancer events to appear, making statistical evidence more meaningful. Second, the patient’s tumor, lymph node, metastasis stage, body mass index, and family history could not be obtained because of the limitation of the NHIRD. This study only explored recordable risk factors based on reliable clinical classifications. Although the distributions of these factors, including covariates, were significantly different between the two groups (patients with and without NSAID therapy), they were well balanced after PSM. Third, on the pathologic subtypes of primary breast cancer was not available. We assumed indirectly that patients who ever received hormone therapy were estrogen-receptor or progesterone-receptor positive, and those who received target therapy had human epidermal growth factor receptor 2 overexpression. The triple negative breast cancer might have been missed in this type of analysis. Finally, a causal relationship between NSAID consumption and second cancer risk could not be inferred directly owing to the observational nature of our study.

In conclusion, with a large collection of approximately 10,000 patients with breast cancer in Taiwan, this population-based cohort study showed that those taking non-COX-2 inhibitors and those receiving multiple NSAIDs during follow-up had a lower second cancer incidence in a dose-dependent manner. To the best of our knowledge, this study is the first to investigate the relationship between NSAID use and second cancer incidence in patients with primary breast cancer. Studies with a prospective design, larger sample size, and longer follow-up period are needed to further verify our findings.

## Data Availability Statement

The data set for this article are not publicly available because public availability of the data set is restricted by local regulations to protect privacy. Any researcher interested in accessing the data set can submit an application form to the Ministry of Health and Welfare requesting access.

## Ethics Statement

This study was approved by the Research Ethics Committee of China Medical University (CMUH-104-REC2-115-R4), and the need for informed consent was waived. Written informed consent for participation was not required for this study in accordance with the national legislation and the institutional requirements.

## Author Contributions

Study concept and design: Y-CL and S-HW. Acquisition of data: S-HW and Y-JP. Analysis and interpretation of data: all authors. Drafting of the manuscript: Y-CL, S-HW, and Y-JP. Critical revision of the manuscript for important intellectual content: all authors. Statistical analysis: S-HW, M-CL, and C-CL. Obtaining funding: Y-CL, S-HW, and Y-JP. Administrative, technical, or material support: Y-CL, S-HW, and Y-JP. Study supervision: S-HW and Y-JP. All authors contributed to the article and approved the submitted version.

## Funding

This study was supported by Ditmanson Medical Foundation Chia-Yi Christian Hospital (Grant No. R104-9), China Medical University Hospital, Taiwan (DMR-109-161), China Medical University, Taiwan (CMU108-MF-62; CMU108-S-23; CMU109-MF-111; CMU110-S-22), Taiwan Ministry of Health and Welfare Clinical Trial Center (MOHW109-TDU-B-212-114004), MOST Clinical Trial Consortium for Stroke (MOST 109-2321-B-039-002), and Tseng-Lien Lin Foundation, Taichung, Taiwan.

## Conflict of Interest

The authors declare that the research was conducted in the absence of any commercial or financial relationships that could be construed as a potential conflict of interest.

## Publisher’s Note

All claims expressed in this article are solely those of the authors and do not necessarily represent those of their affiliated organizations, or those of the publisher, the editors and the reviewers. Any product that may be evaluated in this article, or claim that may be made by its manufacturer, is not guaranteed or endorsed by the publisher.
