# Supplementary figures and images for: Nonsteroidal Anti-Inflammatory Drugs Reduce Second Cancer Risk in Patients With Breast Cancer: A Nationwide Population-Based Propensity Score-Matched Cohort Study in Taiwan
Source: Front Oncol. 2021 Nov 24;11:756143. doi: 10.3389/fonc.2021.756143 (PMC8651993; doi:10.3389/fonc.2021.756143)

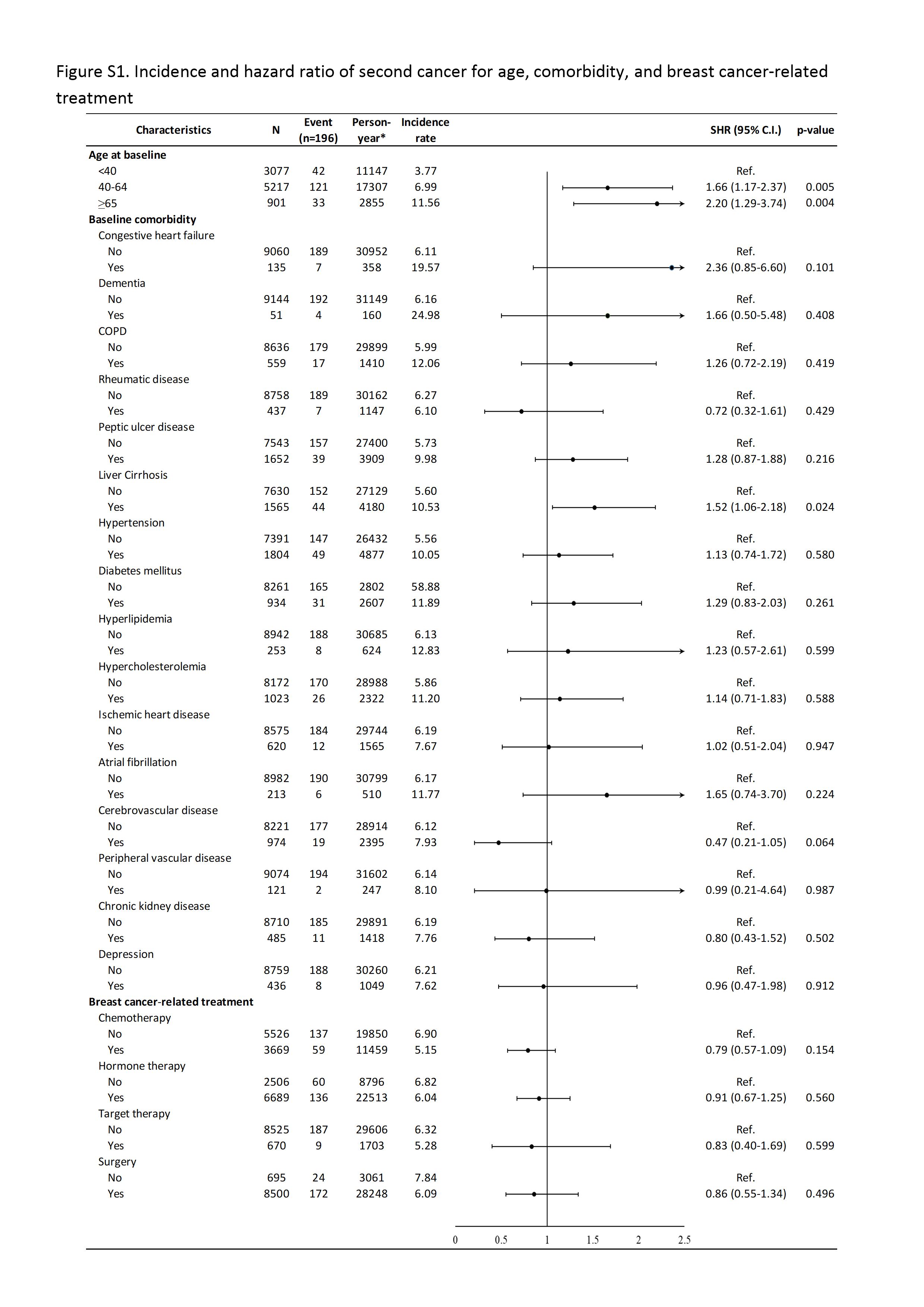

Supplement: Supplementary file 1 [file Image_1.jpeg]
